# Paraneoplastic Cerebellar Degeneration Associated with Breast Cancer: A Case Report and a Narrative Review

**DOI:** 10.3390/brainsci14020176

**Published:** 2024-02-14

**Authors:** Rosario Luca Norrito, Maria Grazia Puleo, Chiara Pintus, Maria Grazia Basso, Giuliana Rizzo, Tiziana Di Chiara, Domenico Di Raimondo, Gaspare Parrinello, Antonino Tuttolomondo

**Affiliations:** U.O.C di Medicina Interna con Stroke Care, Dipartimento di Promozione della Salute, Materno-Infantile, di Medicina Interna e Specialistica di Eccellenza “G. D’Alessandro”, University of Palermo, 90127 Palermo, Italy; rosario94.norrito@gmail.com (R.L.N.); dott.ssamgpuleo@gmail.com (M.G.P.); chiara.pintus@unipa.it (C.P.); mariagrazia.basso@libero.it (M.G.B.); giulianarizzo@yahoo.it (G.R.); tiziana.dichiara@unipa.it (T.D.C.); domenico.diraimondo@unipa.it (D.D.R.);

**Keywords:** paraneoplastic neurological syndromes, malignancy, autoimmunity, antibodies

## Abstract

Paraneoplastic neurological syndromes (PNSs) are an uncommon complication of cancer, affecting nearby 1/10,000 subjects with a tumour. PNSs can involve all the central and peripheral nervous systems, the muscular system, and the neuromuscular junction, causing extremely variable symptomatology. The diagnosis of the paraneoplastic disease usually precedes the clinical manifestations of cancer, making an immediate recognition of the pathology crucial to obtain a better prognosis. PNSs are autoimmune diseases caused by the expression of common antigens by the tumour and the nervous system. Specific antibodies can help clinicians diagnose them, but unfortunately, they are not always detectable. Immunosuppressive therapy and the treatment of cancer are the cornerstones of therapy for PNSs. This paper reports a case of PNSs associated with breast tumours and focuses on the most common paraneoplastic neurological syndromes. We report a case of a young female with a clinical syndrome of the occurrence of rigidity in the right lower limb with postural instability with walking supported and diplopia, with a final diagnosis of paraneoplastic cerebellar degeneration and seronegative rigid human syndrome associated with infiltrating ductal carcinoma of the breast.

## 1. Background

Paraneoplastic neurological syndromes are rare diseases correlated with cancer but not directly caused by the tumour or its complications, such as metabolic deficiencies, side effects of treatment, or coagulopathies [1]; they are capable of involving any part of the nervous system, the muscular system, and the neuromuscular junction. Small cell lung cancer (SCLC), followed by breast and gynaecologic tumours, are most commonly linked to PNSs [2]. However, neurological symptomatology often precedes the clinical manifestations of cancer, making the diagnosis a significant challenge for clinicians; consequently, early recognition of PNSs can hugely influence the prognosis of the neoplastic disease.

The identification of some antibodies, called “onconeural antibodies”, against antigens expressed by both the central nervous system (CNS) and some cancers has been sustained by some researchers as a hypothesis that PNSs are autoimmune diseases [3]; nevertheless, autoantibodies are found in less than 50% of the patients affected by PNSs [4].

A critical insight into the pathogenesis of the disorders was the recognition by Jerome Posner and coworkers that patients with PNSs harbour high-titer antibodies in both their serum and spinal fluid that recognize apparently identical antigens in Western blots of normal brain and tumour tissues of PNSs [4].

In PNSs, an immune response against an underlying systemic tumour is misdirected to the nervous system, causing the clinical manifestations [4]. Most PNSs associate with onconeural antibodies against intracellular antigens [4]. Since their initial description, it has been acknowledged that onconeural antibodies could occur in 5–15% of patients without cancer or in cancer patients without PNSs [3,4].

The updated criteria include novel phenotypes and immune-mediated pathogenic mechanisms, identified since 2004, that emphasize a causal (and not merely chronological) association with cancer and require the demonstration of neuronal antibodies using gold-standard techniques. These three elements represent the core of the present criteria of PNSs [5,6]. These novel criteria substituted “classical syndromes” with the term “high-risk phenotypes” for cancer and introduced the concept of “intermediate-risk phenotypes” and replaced the term “onconeural antibody” by “high risk” (>70% associated with cancer) and “intermediate risk” (30–70% associated with cancer) antibodies. The new criteria also indicated three levels of evidence for PNSs: definite, probable, and possible [5,6]. In 2022, a Chinese evaluation of the updated criteria for PNSs emphasized the consistency between cancer phenotype and antibody and showed a better diagnostic value. A better diagnostic yield could benefit disease management [7].

Despite their associated human and economic costs, these neuroimmune disorders have seldom been the subject of epidemiologic studies. The annual incidence per million person years has been estimated at 8.9 for PNSs in Northeastern Italy, 5 for antibody-positive AE in Olmsted County, MN, 0.83 for leucine-rich glioma inactivated 1 (LGI1) encephalitis in the Netherlands, and 0.9 to 2.2 for paediatric N-methyl-D-aspartic acid (NMDAr) encephalitis in the United Kingdom and Hong Kong. All of these studies have reported year-to-year increases in incidence in a context of increased diagnostic abilities and improved recognition of clinical syndromes [8].

Once the diagnosis is established, it is necessary to start an aggressive immunosuppressive therapy and a specific treatment for the underlying cancer as soon as possible.

Here, we report a case of a paraneoplastic neurological syndrome associated with breast cancer and a narrative review that will focus on the most common paraneoplastic neurological syndromes, including limbic encephalitis, neuronopathies, cerebellar degeneration, dermatomyositis, chronic gastrointestinal pseudo-obstruction, opsoclonus-myoclonus, and peripheral nerve hyperexcitability syndromes.

## 2. Case Description

We report the case of a 36-year-old woman presenting the thalassemia trait who reported in February 2020 the onset of rhinorrhoea, conjunctival haemorrhage in the right eye and subsequent appearance of paresthesias of the ipsilateral eyelid, and subsequent painful symptoms with a jolting sensation along the course of the trigeminal branch, first in the right half of the face and subsequent involvement of the left half of the face. She also complained of a recurrent pulsating headache of severe intensity associated with postural instability treated with oral pregabalin 75 mg trice a day with the improvement of symptoms. For this reason, she also underwent a brain MRI which showed no structural alterations.

In May 2021, she complained of paraesthesia and itching sensation in the lumbosacral region and some episodes of urinary retention, so in May 2020, she undertook a cervical MRI which revealed only the presence of a herniation of the C5–C6 intervertebral disc.

In June 2020, she reported the onset of transient diplopia lasting about two days. Then, she performed the Hess Lancaster test, which showed hypofunction of the right lower rectum muscle and a regular field test.

In July 2020, the patient complained of a recurrence of diplopia associated with ptosis of the right eyelid and subsequent appearance after a few days of rigidity of the right lower limb, especially when climbing stairs and difficulty in lifting it.

She performed chest CT which showed minimal thymic hyperplasia, for which a neurologist suggested a possible diagnosis of Myasthenia Gravis. Therefore, the neurologist prescribed therapy with pyridostigmine bromide and prednisone with immediate benefit demonstrated by the reduction in the visual disturbance and the right lower limb stiffness. The patient also performed electrical and mechanical responses to single shocks, slow and fast nerve stimulation (RNS), quantitated EMG, anti-acetylcholine receptor (AChR), and anti-striated muscle (SM) antibodies (ab) that reported no pathological finding.

In summer 2020, she underwent a new neurological consult that definitively excluded the Myasthenia Gravis’s suspicion, indicating the withdrawal of steroid and pyridostigmine bromide therapy; it also suggested a possible alternative diagnosis of previous multiple cranial mononeuropathies.

In September 2020, the patient complained of the recurrence of ataxia of the right lower limb with postural instability and walking supported by the use of a cane. Therefore, the patient experienced a new recurrence of diplopia. She also reported a fall due to postural instability with multi-fragmented displaced fracture of the distal radius epiphysis and ulnar styloid of the left wrist. The neurologist indicated an autoimmunity screening with immunofluorescence assay with a positive antinuclear antibody (ANA) and weak positive anti-titin antibody. In the same period, another neurologist suspected a probable neuroinflammatory disease and, on this basis, an anti-neurone antibody dosage was planned. She also underwent another brain MRI which showed a few minutes nonspecific gliotic lesions in the right frontal cortical juxtacortical site and the deep white matter adjacent to the posterior section of the ipsilateral lateral ventricle with no pathological enhancement after contrast medium. In the same period, the patient underwent diagnostic lumbar puncture (LP) with normal cerebrospinal fluid (CSF) findings, including appearance, opening pressure, WBC count, glucose level, protein level, immunoglobulins (IgG, IgM, IgA), and albumin. Isoelectric focusing of paired CSF and serum samples was used to identify CSF-specific oligoclonal IgG bands (OCB) with negative findings.

Therefore, she was admitted to the Ward of Neurology of the University Hospital of Palermo on the last days of September 2020. At the admission to this ward, she had a normal-holding upright position with multidirectional oscillations, uncertain gait, right eyelid ptosis, left cranial nerve VI deficit, diplopia in the lateral gaze towards the left, multi-directional rotary nystagmus prevalent in the lateral gaze to the left, mixed hypertonus with a prevalent spastic component of the right lower limb, right Babinski, and mandibular deviation to the right. She underwent high-dose intravenous methylprednisolone bolus therapy of 1 g daily for five days.

This neurological picture, characterized by eyelid ptosis, diplopia, and nystagmus, could suggest a cerebellar involvement. In the light of a possible ischemic aetiology, the patient underwent MRI and brain CT with a contrast medium which excluded recent ischemic lesions and abnormalities or stenosis of the intra and extracranial vessels.

On the first days of October 2021, because of the closure of the Neurology Ward due to the redistribution of the hospital wards due to the SARS-CoV2 recrudescence, the patient was transferred to our Internal Medicine and Stroke Care Ward. At admission, the patient was in poor clinical condition. She was alert, cooperative, eupnoeic at rest, and apyretic. The physical examination performed at the entrance showed slight left eyelid ptosis, horizontal “up-looking” nystagmus, and segmental strength deficit measured by means of the Medical Research Council scale for individual muscle groups showing upper limbs 4/5 proximal and 3/5 distal, right lower limb 3/5 proximal 2/5 distal, and left lower limb 3/5 proximal and distal; the deep tendon reflexes appeared to be normoelicitable with a tendency to hyperexcitability. Furthermore, no sensitivity abnormality was present, and a mandibular deviation to the right was observed.

Due to the neurological findings suggestive of truncal and cerebellar involvement and after the exclusion of other ischemic or infective possible pathogenesis, the paraneoplastic pathogenesis seemed reasonable. In particular, the symptomatology was compatible with the paraneoplastic cerebellar ataxia, also known as paraneoplastic cerebellar degeneration, and seronegative rigid human syndrome. These symptoms are common in patients with brainstem paraneoplastic encephalopathy, with reported symptoms of right eyelid ptosis, left cranial nerve VI deficit, and diplopia in the lateral gaze towards the left; nevertheless, these ocular symptoms could be related to cerebellar involvement due the multiple connections between the cerebellum and oculomotor and vestibular nuclei.

For this reason, a whole-body bone SPET/CT scan was performed and showed the presence of tissue with high metabolic activity in the lower-external quadrant of the left breast and of some lymph nodes in the ipsilateral axillary area. Thus, the patient underwent mammography and total body CT with a contrast medium, which confirmed the presence of a 9 mm lesion with post-contrast enhancement in the left breast and some enlarged lymph nodes, the largest of which had a diameter of about 2 cm and was partially colliquated, in the ipsilateral axillary area with the same contrasting characteristics. The breast formation was subsequently biopsied and classified as infiltrating ductal carcinoma G2, ER + (100%), Prog + (50%), Her2- with Ki67 equal to 80%, E-cadherin +. Onconeural antibodies were negative.

Thus, the final diagnosis was a diagnosis of paraneoplastic cerebellar degeneration and seronegative rigid human syndrome associated with infiltrating ductal carcinoma of the breast.

## 3. Discussion

Breast neoplasms and those of gynaecological relevance and small cell lung cancer appear to be among the solid neoformations most frequently associated with paraneoplastic syndromes with neurological involvement. The spectrum of these paraneoplastic pathologies is vast and can involve the brain, cerebellum, spinal cord, and peripheral nerves, and they can present nuanced symptomatology with a sub-acute course.

The patient’s clinical picture excluded limbic encephalitis, characterized by alterations in mood, memory, and psychosis, sub-acute sensory neuropathy, and opsoclonus-myoclonus syndrome, characterized by involuntary contractions of striated muscles and uncontrolled eye movements.

Eyelid ptosis, nystagmus, and diplopia, on the other hand, appeared strongly suggestive of paraneoplastic cerebellar degeneration, which appears to be the most frequent paraneoplastic syndrome in breast cancer patients, although not presenting all the characteristics such as signs of cerebellar atrophy on brain MRI, CSF lymphocytosis, and the presence of anti-neuron antibodies, thus suggesting that it is still an early stage. The antibodies most associated with breast cancer are anti-Yo, anti-Hu, and anti-Ri. While the first two are closely associated with neuronal death resulting in poor prognosis, anti-Ri is related to a better therapeutic response with a greater possibility of restitutio ad integrum [3].

Although, as already specified, breast neoplasms, small cell lung tumours, and gynaecological neoformations are those most associated with paraneoplastic syndromes with neurological involvement, particularly with cerebellar degeneration, thymoma, testicular neoplasms, and Hodgkin’s lymphoma are also significantly associated with this pathology; in fact, other antibodies of possible finding are anti-CV2, related to thymoma and microcytoma, anti-Ma2, associated with testicular malignancy, and anti-Tr, present in patients with Hodgkin’s lymphoma [4].

A separate mention should be made for anti-TG antibodies, associated with gluten sensitivity, and anti-GAD65 antibodies, associated with type 1 diabetes mellitus and whose cerebellar degeneration is typical in patients beyond the fifth decade of life. In addition, numerous other antibodies are being studied to establish and better characterize their association with paraneoplastic cerebellar degeneration, among them PCA-2, Anti-mGluR1, Anti-Tr PCD, Anti-Ca/ARHGAP26, Anti-CARP VIII, Anti-PKCγ, Anti-Nb/AP3B2, Anti-Sj/ITPR1, Anti-CASPR-2, and Anti-neurochondrine.

Although there are no international guidelines regarding paraneoplastic cerebellar degeneration, the literature indicates that the presence of a malignant neoplasm should not be a reason for a delay in undertaking even aggressive induction therapy, specifically methylprednisolone therapy set at a high dosage (1000 mg/day) in association first with plasmapheresis and then with intravenous infusion of immunoglobulins for five days [4].

After this therapy, the patient experienced a rapid and progressive almost complete disappearance of the cerebellar symptoms characterized by diplopia, nystagmus, and eyelid ptosis; this response to therapy would seem to preclude the possible presence of anti-Hu and anti-Yo antibodies, characterized by an absence or low susceptibility to immunosuppressive therapy, favouring the form mediated by anti-Ri antibodies, even if not found.

Symptoms characterized by rigidity in the right lower limb without sensory involvement, segmental strength deficit mainly in the lower limbs, and mandibular deviation to the right, even though the patient presented concomitant structural alterations in the temporomandibular joint, contextualized in the framework of paraneoplastic syndromes are part of the differential diagnosis between paraneoplastic peripheral nerve hyperexcitability and rigid man syndrome. The first is characterized by cramps, pseudothetany, and stiffness in the limbs with a typical electromyographic pattern; the second has similar but more nuanced symptomatology, with the possible presence of muscle weakness and possibly involving the neck and facial muscles. It is strongly associated with breast cancer and electromyography may be within normal limits. For these reasons, the latter appears to be the most likely diagnosis.

Stiff man syndrome can be associated with anti-GAD antibodies, related to symptoms affecting the lower limbs only, and anti-amphysine, responsible for the symptoms also affecting the abdominal, facial, and neck muscles [3]. Nevertheless, these symptoms are common in patients with brainstem paraneoplastic encephalopathy

In light of the high dose of corticosteroid currently practised and subsequent tapering, we have chosen to evaluate the possible introduction of an additional immunosuppressant after an interview with the patient’s oncologist colleagues, discussing jointly neoadjuvant and immunosuppressive therapy that the patient will have to sustain. Therefore, the patient resigns with the momentary indication to practice prednisone exclusively, postponing the possible introduction of a second immunosuppressive agent after the interview as mentioned earlier with oncologist colleagues.

The patient has been discharged with an improved clinical condition and the indication to follow the post-discharge therapeutic programme in an oncology clinical setting.

### 3.1. Paraneoplastic Neurological Syndromes

#### 3.1.1. Pathogenesis

The better-known pathogenic mechanisms of PNSs are the antibody-mediated one and the T-cell-related one.

#### 3.1.2. Association between Antibodies and PNSs

Onconeural antibodies (Table 1) have high specificity for neoplastic disease, and their presence in subjects with suggestive neurological symptomatology can confirm a diagnosis of PNSs [4].

These antibodies are usually associated with a specific type of cancer; however, it is possible to observe rare exceptions. In fact, Gastaldi et al. and Greenlee et al. reported the presence of ZIC4 and CV2 antibodies in gynaecological tumours, even though they are usually correlated with SCLC [5,6].

As far as it concerns the function of onconeural antibodies in the process of PNSs, Greenlee et al. observed the death of cerebellar Purkinje cells exposed to anti-Yo antibodies [9]; in addition, anti-amphiphysin antibodies seem to cause rigidity and muscular contractions in rats by inhibiting the release of γ-aminobutyric acid (GABA) [9]. The pathogenic role of the other onconeural antibodies is still unclear, but they might not directly influence PNSs’ development. In fact, these antibodies can be found in subjects with tumours but not a paraneoplastic disease, and multiple tries to create animal models have failed.

Other antibodies directed against surface antigens of various brain cytotypes are correlated to the appearance of neurological symptoms, but they are not specific to paraneoplastic aetiology and do not have a strict association with a specific tumour (Table 1).

These antibodies seem to play a direct pathogenic role; in fact, the seriousness of the symptomatology is usually proportional to the antibody titers and decreases after immunosuppressive therapy.

**Table 1:** Antibodies associated with PNSs.

Antibody	Neurological Findings	Tumor Associated	Epidemiological Data
Hu (ANNA-1) [10,11,12].	SNN, chronic gastrointestinal, pseudo-obstruction, EM, and LE	SCLC >> NSCLC, other neuroendocrine tumors, and neuroblastoma	Patients aged <18 Y
CV2/CRMP5 [13,14,15,16,17].	EM and SNN	SCLC and thymoma	Patients with an associated thymoma are younger
SOX1 [18].	LEMS with and without rapidly progressive cerebellar syndrome	SCLC	Association with SCLC than with a particular neurologic presentation
PCA2 (MAP1B)	Sensorimotor neuropathy, rapidlyprogressive cerebellar syndrome, and EM	SCLC, NSCLC, and breast cancer	
Amphiphysin	Polyradiculoneuropathy, SNN, EM, SPS	SCLC and breast cancer	Women with breast cancer and SPS
Ri (ANNA-2) [19].	Brainstem/cerebellar syndrome, OMS	Breast > lung (SCLC and NSCLC)	Breast cancer in women; lung cancer in men
Yo (PCA-1) [20].	Rapidly progressive cerebellar syndrome	Ovary and breast cancers	More frequent in women
Ma2 and/or Ma [21,22,23].	LE, diencephalitis, and brainstem encephalitis	Testicular cancer and NSCLC	Young men → testicular tumors
Tr (DNER) [17].	Rapidly progressive cerebellar syndrome	Hodgkin lymphoma	
KLHL11 [18,19,24].	Brainstem/cerebellar syndrome	Testicular cancer	Young men
AMPAR [25,26].	Limbic encephalitis	SCLC and malignant thymoma	
GABABR [26].	Limbic encephalitis	SCLC	Men, smokers
mGluR5 [27].	Encephalitis	Hodgkin lymphoma	
P/Q VGCC [28,29,30,31].	LEMS, rapidly progressive cerebellar syndrome	SCLC	
NMDAR [32,33,34,35].	Anti-NMDAR encephalitis	Ovarian or extraovarian teratomas	Females aged between 12 and 45 y (50%). Elderly patients have less frequent tumors (<25%)
mGluR1 [15,36,37,38]	Cerebellar ataxia	Mostly hematologic	
GABAAR [22].	Encephalitis	Malignant thymoma	Adults (60%)
CASPR2 [25,39,40].	LE, acquired neuromyotonia (Isaac syndrome), and Morvan syndrome	Malignant thymoma	Associated (≈50%) with malignant thymoma
GAD65 [25,41,42,43]	LE, SPS, and cerebellar ataxia	SCLC, other neuroendocrine tumors, and malignant thymoma	Elderly
DPPX [25].	Encephalitis with CNS hyperexcitability and PERM	B-cell neoplasms	
GlyR [1,2,25]	LE and PERM	Malignant thymoma and Hodgkin lymphoma	

Abbreviations: AMPAR = α-amino-3-hydroxy-5-methyl-4-isoxazolepropionic acid receptor; ANNA = antineuronal nuclear antibody; CASPR2 = contactin-associated protein-like 2; CRMP5 = collapsin response-mediator protein 5; DNER = delta/notch-like epidermal growth factor-related receptor; DPPX = dipeptidyl peptidase-like protein; EM = encephalomyelitis; GABAAR = gamma-aminobutyric acid-A receptor; GABABR = gamma-aminobutyric acid-b receptor; GAD = glutamic acid decarboxylase; GlyR = glycine receptor; KCTD16 = potassium channel tetramerization domain-containing; KLHL11 = Kelch-like protein 11; LE = limbic encephalitis; LEMS = Lambert–Eaton myasthenic syndrome; MAP1B = microtubule-associated protein 1B; mGluR1 = metabotropic glutamate receptor type 1; mGluR5 = metabotropic glutamate receptor type 5; NMDAR = NMDA receptor; NSCLC = non-small cell lung cancer; OMS = opsoclonus-myoclonus syndrome; PCA = Purkinje cell antibody; PERM = progressive encephalomyelitis with rigidity and myoclonus; SCLC = small cell lung cancer; SNN = sensory neuronopathy; SPS = stiff-person syndrome; VGCC = voltage-gated calcium channel.

The development of new and more effective methods has helped researchers to discover new antibodies. As far as it concerns limbic encephalitis, the antibodies anti-GABA_B_ receptor [7] and anti-AMPA receptor [44] were found in all the subjects affected by SCLC and Hu-negative paraneoplastic limbic encephalitis. In addition, Lancaster et al. reported the presence of antibodies directed against the glutamate receptor 5 (mGluR5) in patients affected by Ophelia syndrome, with an association between limbic encephalitis and Hodgkin’s disease [45].

Anti-NMDAR antibodies are usually correlated with encephalitis in subjects affected by ovarian teratoma and less frequently by other neoplasms [32].

It is also possible to observe the production of IgM or IgG directly by the tumour, typically in subjects affected by Waldenström’s macroglobulinemia and lymphomas. In particular, these neoplasms are associated with peripheral sensorimotor and motor neuropathy, producing antibodies against antigens of the nerve. However, the pathogenic mechanism is still poorly understood [33,34].

#### 3.1.3. Role of T-Cells in PNSs

**Figure 1:**
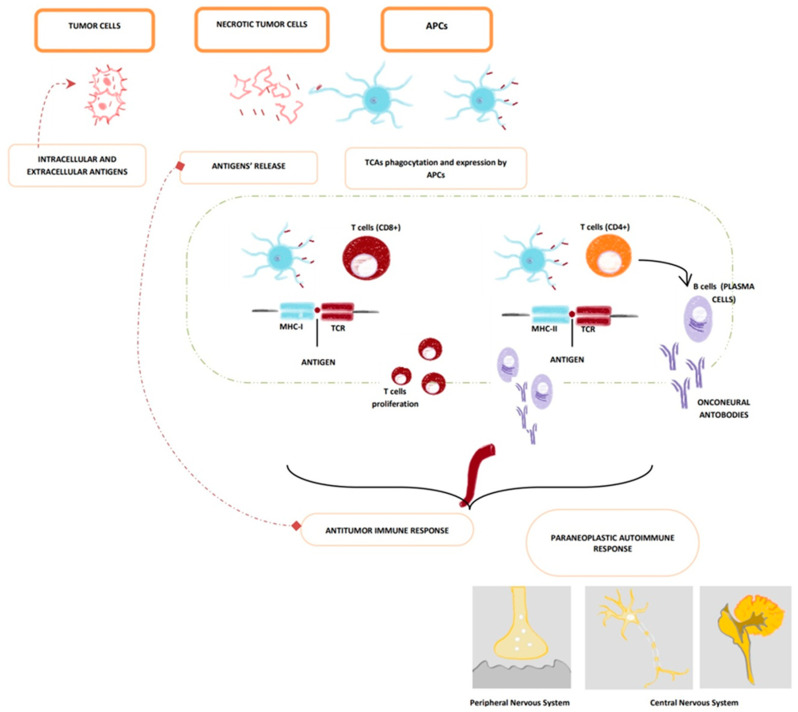
Pathogenetic cross reactions in pathogenesis of PNSs (paraneoplastic neurological syndromes). Common antigens of the tumour and the nervous system are presented to the immune system through tumour cells’ necrosis which leads to antigens’ release and phagocytation and presentation by APCs (Antigen-Presenting Cells), which migrate towards the lymph node area (illustrated in the figure above by the green dotted line). In nodes, APCs present tumour cells’ antigens to CD8+ cytotoxic T cells and CD4+ t-helper T + cells (thus leading to plasma cells activation and antibody production). Due to antigens’ cross-reactivity, this cross reaction induces the production of CD8+ cytotoxic T cells and autoantibodies that target the nervous system (both peripheral nervous system and central nervous system. APCs (Antigen-Presenting Cells); TCAs (Tumour Cell Antigens); MHC-I (Major Histocompatibility Complex—class I); MHC-II (Major Histocompatibility Complex—class II); TCR (T-Cells Receptor).

T cells seem to have a crucial role in the development of PNSs (Figure 1). Dalmau et al. observed a considerable amount of CD4+ and CD8+ cells in the cerebral areas of the central nervous system affected by PNSs of the central nervous system (CNS) [46]. Since neurons do not express the antigen-presenting molecules MHC class I and II in normal conditions, the process of recognition between T cells and neurons is still unclear. A plausible mechanism is the expression of MHC molecules by neurons after an injury [47]; the consequent inflammation could help T cells reach the cerebral parenchyma thanks to pro-inflammatory cytokines, chemokines, and adhesion molecules, especially ICAM-1 [48]. In addition, Dalmau et al. observed that SCLC of patients with concomitant PNSs had significantly higher titres of MHC molecules of class I and II compared to the ones without PNSs [49]. Consequently, it is plausible that there is a possible connection between the antigens expressed by the tumour and the ones expressed by the CNS that stimulates the hyperactivation of the immune system responsible for the neurological symptomatology.

A massive presence of T-cells has been observed in patients affected by PNSs, mediated by anti-Ta [50], 19anti-Ri [50], anti-Hu [51], anti-Ma antibodies [52].

On the other hand, considerable research [26,53,54] has reported a reduced infiltration of mononuclear cells in the CNS of subjects affected by cerebellar degeneration associated with anti-Yo antibodies, partially justifying the better prognosis of PNSs mediated by this antibody compared to the other ones.

Thanks to immunohistopathology, it was observed that the T-cells CD3+ and CD8+ are the most numerous cytotypes in cerebral areas affected by PNSs [55]; CD8+ causes cell injury and death, secreting perforin and granzyme-B. Other identified mechanisms are the activation of the Fas-FasL pathway and the tumour necrosis factor (TNF) receptor [56].

Other cell types spotted in the cerebral areas involved in the paraneoplastic disease, especially during the first phase, are macrophages, CD68+ [57], B-cells, and plasma cells; T-cells CD4+ represent a minor component [58].

In subjects affected by PNSs, regulatory T-cells (T_reg_) seems to be suppressed, as demonstrated by the reduced expression of TGF-β, FOXP3, and CTLA4 [59].

The cerebrospinal fluid (CSF) of patients affected by CNS is often characterised by oligoclonal bands and pleocytosis, especially during the first steps of the disease [60]. The most expressed cytotypes are B-cells and CD8+/CD4+ T-cells, and NK cells are a minor component [61].

#### 3.1.4. Paraneoplastic Limbic Encephalitis

Paraneoplastic limbic encephalitis (PLE) is a rare complication of neoplastic disease. The neurological symptomatology precedes cancer diagnosis in almost 60% of the cases [62]. SCLC is most frequently associated with PLE, followed by testicular tumours, breast cancer, and Hodgkin’s disease [62].

Multiple antibodies are correlated to PLE, and they are usually associated with a specific tumour (Table 2). The type of antibody also influences the prognosis; anti-Hu is one of the most common and is correlated to a poor prognosis. In the case of anti-VGKC, anti-NMDA-R, and anti-Yo, the outcome seems to be better instead [20].

The development of the symptomatology is usually subacute; the typical clinical presentation is characterised by amnesia, seizures, and rapidly progressive dementia; other symptoms are confusion, depression, and hallucinations, and it is also possible to observe hypothalamic impairment defined by panhypopituitarism and hyperthermia [63].

Brain MRI has a low sensibility (nearby 50%), but possible findings can be hyperintensity in T2 or FLAIR signal typically in medial temporal lobes [34]; on the other hand, fluorodeoxyglucose positron emission tomography (PET) seems more sensitive than MRI, showing hypermetabolic activity in these areas [64]. EEG shows some aspecific alterations characterised by disorganised and slow waves, especially during seizures [65].

As far as it concerns the CSF, lymphocytic pleocytosis is observed in the majority of the cases; other common findings are hyperprotidorrachia (>45 mg/dL) and the presence of oligoclonal bands and IgG. Malignant cells are not usually identified in the CSF [63].

The differential diagnosis includes metabolic encephalopathies, neurodegenerative pathologies (Alzheimer’s disease, Parkinson’s plus), infectious encephalitis, vasculitis of the CNS, and stroke.

To conclude, every cerebral area could be potentially involved in the paraneoplastic disease, leading to variable neurological symptomatology; the brainstem and the cerebellum are commonly involved and overlapping syndromes are possible.

#### 3.1.5. Limbic Encephalitis Associated with Anti-Neuronal Nuclear Antibody, Type I (Anti-Hu)

Multiple intracellular RNA binding proteins are the target of anti-Hu antibodies, and SCLC expresses one of these intracellular molecules called HuD [10].

Apart from limbic encephalitis, other neurological symptoms correlated to this antibody are characterised by motor neuropathy, autonomic dysfunction, brainstem and cerebellar involvement, aphasia, and visual field alterations [11]. Most subjects with this antibody have SCLC, but anti-Hu is detected only in half of the patients affected by this cancer and limbic encephalitis [12].

Anti-Hu-related PNSs usually have a poor outcome; the treatment of the underlying neoplasm could stop the progression of the neurological disease, but restitutio ad integrum is uncommon.

#### 3.1.6. Limbic Encephalitis Associated with N-Methyl-D-Aspartate Receptor (NMDAR) Antibodies

NMDAR antibodies are usually associated with ovarian teratomas, which are benign in nearly 65% of the cases, and they are supposed to alter the neural connection.

The peculiarity of NMDAR-associated encephalitis is the common presence of psychiatric symptomatology ascribable to schizophrenia, leading to a significant diagnosis delay [66]; other reported symptoms include amnesia, seizures, dyskinesias, autonomic dysfunctions, alterations of the consciousness, and depression of the respiratory drive [35].

Brain MRI is often normal, but it is possible to observe some alterations in FLAIR and T2 sequences, typically in medial temporal lobes, the brainstem, and the cerebellum. The CSF is usually characterised by pleocytosis with a prevalence of lymphocytes and oligoclonal bands [35].

The removal of the neoplasms, combined with immunosuppression, is crucial for the regression of the symptomatology.

#### 3.1.7. Limbic Encephalitis Associated with Voltage-Gated Potassium Channel (VGKC) Antibodies

VGKC antibodies are usually associated with prostate cancer, SCLC, and thymoma. However, no cancer was detected in most patients with these antibodies [28,29]. The development of neurological symptomatology is typically subacute and is characterised by amnesia, seizures, and REM sleep alterations; hyponatraemia is quite common [67,68]. MRI can show alterations of the amygdala, hippocampus, and basal ganglia [69]; the pleocytosis observed in the CSF is milder than in other paraneoplastic encephalitides [67].

#### 3.1.8. Limbic Encephalitis Associated with Amphiphysin Antibodies

Amphiphysin antibodies are typically observed in patients affected by SCLC and breast cancer; however, these antibodies are rarely associated with limbic encephalitis. It is usually possible to observe the simultaneous presence of anti-VGKC and anti-Hu, especially in subjects with SCLC [70].

These antibodies are directed against two types of antigens: (1) intracellular antigens (Hu, Ma2, CRMP5, amphiphysin, etc.) and (2) cell surface antigens (the VGKC complex, AMPARs, GABABRs, mGluR5 receptor, GlyRs, etc.). Whereas the first type of antibodies have been reported as associated with cancer (lung, testis, etc.) and high a degree of cytotoxic T cells infiltrating the brain, the second type of antibodies have been reported as associating less frequently with cancer (thymoma, teratoma) and are antibody mediated. These two antibodies have in common the association with idiopathic or paraneoplastic limbic encephalitis [M. Lai, E. G. Hughes, X. Peng et al., “AMPA receptor antibodies in limbic encephalitis alter synaptic receptor location,” Annals of Neurology, vol. 65, no. 4, pp. 424–434, 2009 [7], E. Lancaster, M. Lai, X. Peng et al., [26] “Antibodies to the GABAB receptor in limbic encephalitis with seizures: case series and characterisation of the antigen”, The Lancet Neurology, vol. 9, no. 1, pp. 67–76, 2010 [71]].

#### 3.1.9. Limbic Encephalitis Associated with Anti-Ma2 Antibodies

The family of Ma proteins is involved in DNA transcription and is typically expressed in testicular germ cells and neurons; Ma2 is the most represented of these proteins [72]. Antibodies against Ma2 are generally detected in subjects affected by testicular germ cancer, breast cancer, and SCLC [21].

The peculiarity of this encephalitis is the simultaneous involvement of the hypothalamus and the brainstem. The hypothalamic impairment is characterised by diabetes insipidus, REM sleep alterations, hyperthermia, and hypersomnia [22]; on the other hand, cerebellar ataxia and vertical gaze paresis indicate brainstem dysfunction.

Brain MRI usually shows thalamus, medial temporal lobes, and hypothalamus alterations mimicking cancer [21]; in addition, hypocretin in the CSF is commonly absent [23].

#### 3.1.10. Limbic Encephalitis Associated with Collapsin Response-Mediator Protein (CRMP-5) Antibody

This antibody has been detected in subjects affected by PNSs with various neoplasms, typically thymoma and SCLC. However, its presence is not strictly associated with PNSs [13].

The possible presence of chorea characterises limbic encephalitis mediated by anti-CRMP-5 antibodies [14], but in most cases, it is clinically and radiologically similar to the other forms of paraneoplastic limbic encephalitis [73].

### 3.2. Paraneoplastic Cerebellar Degeneration

Paraneoplastic cerebellar degeneration (PCD), also known as paraneoplastic cerebellar ataxia, is one of the most common paraneoplastic neurological syndromes (PNSs) [74,75]. Although available epidemiological data are limited, studies report that it approximately affects 1% of patients with cancer [76] and is considered the most common immune-mediated cerebellar ataxia [77,78,79]. Moreover, recently, a nine-year (2009–2017) population-based study conducted in northern Italy reported an incidence of paraneoplastic syndromes of 0.89/100,000 person years, finding that among 89 patients with definite PNSs, 25 (28.1%) had PCD [79].

PCD is a group of neurological disorders resulting from tumour-induced autoimmunity against cerebellar antigens [80,81] and proteins restricted to immune-privileged neurons, which are then presented by the underlying malignancy [82]. Thus, the immune system produces the so-called “onconeural antibodies” (ONAs), which cross-react with similar proteins expressed inside or outside, as membrane proteins, of cerebellar Purkinje cells, causing their death; hence, cerebellar dysfunction [55,83].

#### 3.2.1. Association between Paraneoplastic Cerebellar Degeneration and Autoantibodies

There are nearly 30 different antibodies associated with PCD [84]; these include anti-Yo (PCA-1), anti-Hu, anti-Ri, anti-Tr, anti-VGCC, anti-Ma, anti-CRMP5 (anti-CV2), and anti-mGluR [27] These antibodies are found in 80% of PCDs [15] and each one of them correlates with specific tumour types [16], thus guiding the search for the causative cancer.

These antibodies are classified according to the cellular localization of their targets (intracellular, cell-surface, or synaptic) [17]. Only a few antibodies are directed against antigens expressed on neuronal membranes. In contrast, most of them have intracellular neuronal protein as a target [36,37].

This classification is probably directly linked to their pathophysiological function, which is still unclear. Autoantibodies directed toward intracellular antigens probably do not determine Purkinje cells’ death. Instead, they may mainly be considered a marker of the autoimmune process [19], and this is proven because histological cerebellum studies have not shown the presence of B cells/IgG or complements but detected CD8+ cells and microglia instead [24]. Moreover, the analysis of cerebrospinal fluid cells via fluorescent-activated cell sorting revealed T cells to be the predominant cell type. In contrast, B cells constituted only a less represented component [18].

On the other hand, antibodies directed against the neural cell surface or synaptic proteins (such as P/Q-type voltage-gated calcium channels (VGCCs) and metabotropic glutamate receptor 1 (mGluR1) antibodies), are thought to have a direct pathogenic role as they link to surface receptors, causing blockage and internalization, which ultimately results in cerebellar ataxia [38]. This assertion is also suggested, for example, by the fact that VGCCs are responsible for calcium homeostasis and play a relevant role in cell function and survival [38]. The binding of VGCC antibodies leads to internalization of the channel and causes premature cell death, as has been demonstrated by autopsy studies of patients with VGCC antibody-associated PCD, which have documented depletion of VGCCs and binding of VGCC antibodies to the remaining channels in a context of diffuse loss of Purkinje cells [85,86].

#### 3.2.2. Classification of PCD

The most common variant of PCD is ataxic syndrome, associated with the Anti-Yo antibody or Purkinje cell cytoplasmic antibody type 1 (PCA1) [18], which is more frequently associated with breast cancer and gynaecological malignancy (tumours of the ovary, endometrium, and fallopian tube) [2,87,88,89]. However, rare findings of anti-Yo antibodies have also been described in lung cancers [90] and adenocarcinomas of the gastrointestinal system and prostate [91,92]. The underlying mechanisms responsible for these antibodies’ production is thought to be linked to the cerebellar degeneration-related protein 2 (CDR2), a protein usually found in the cerebellum that is ectopically produced by tumour cells [18].

Anti-Yo-mediated PCD has the poorest response to standard therapies and the poorest outcome. Rapid Purkinje cell death leads to rapid progression of disability; in the later stages of the disease, less than 10% of patients can ambulate without assistance, with a long-term survival rate less than 25% [89,91,93,94]. Concerning this aspect of progressive disability, early treatment and intense rehabilitation are considered important in impacting the clinical course of PCD [94,95].

Patients affected by PCD can present a variety of symptoms, showing an acute or a subacute time-course onset [96,97,98]. Usually, symptoms resembling a viral infection (dizziness, nausea, vomiting) characterize the early stages. Mostly in a few weeks, they are followed by gait instability, limb and truncal ataxia, and symptoms associated with brainstem involvement such as dysarthria and dysphagia [91,98,99].

The deficits are often bilateral but show some asymmetry [2]. Less common presentations are acute stroke-like presentations, ataxic dysarthria, or episodic vertigo that can initially mimic benign positional vertigo. Many affected individuals have tremor of the trunk and head and a marked high-amplitude intention tremor that causes difficulty in everyday tasks requiring the upper limbs. Communication can be impaired not only because of severe dysarthria but because PCD also causes prosody loss and irregular articulation, speech rate, and volume. The ocular motor abnormalities that accompany pcd can be complex because there is always some degree of brainstem encephalitis that can disrupt either vestibulocerebellar connections or associated ocular brainstem nuclei and they can erroneously evocate a primitive brainstem involvement [19].

The importance of detecting these signs is evident since cerebellar ataxia is the first manifestation of neoplasm in 70% of patients [98].

It is challenging to differentiate PCD from another cerebellar ataxia, as can easily be guessed. Other diseases such as demyelinating diseases (e.g., multiple sclerosis), systemic autoimmune disorders (e.g., sarcoidosis, lupus), metabolic diseases (alcohol abuse, Wernicke’s syndrome, Vitamin E, B12 deficiencies), gluten ataxia (Celiac disease), infectious (e.g., prion diseases) or postinfectious cerebellitis (Guillain-Barre syndrome), and drug-associated toxicity [80] can have a similar clinical picture.

#### 3.2.3. Diagnosis of PCD

Therefore, the diagnostic evaluation begins with laboratory testing (e.g., vitamin levels to exclude metabolic causes or anti-gliadin antibodies to exclude gluten ataxia) and neuroimaging; brain magnetic resonance imaging (MRI) is not sensitive in the initial stages of the disease [88,100,101].

Cerebrospinal fluid analysis (CSF) of PCD patients can show both aspecific inflammatory changes in the early stages, such as primarily elevated protein, lymphocytic pleocytosis, and oligoclonal bands [80], and more specific findings such as 14-3-3 protein, specific for Creuzfield–Jacob diseases, thus leading to a wrong diagnosis, especially if one does not have instrumental findings suggesting the presence of a tumour [102].

After performing these laboratory tests and imaging, after excluding other aetiologies of acute/subacute cerebellar ataxia, a paraneoplastic antibody panel on a blood sample must be conducted.

If ONAs are detected, it is crucial to search for a primary occult malignancy using a whole-body computed tomography (CT) scan as a first-level analysis and a successive positron emission tomography (PET) scan if needed [103]. If the initial tumour screen is negative, patients should be followed up with scans at arranged intervals [103]. On the other hand, if ONAs are not detected and clinical suspicion is high, repeating antibody testing is advisable [104]. Antibody-detection techniques (e.g., immunoprecipitation, immunofixation, and immune blots) are not 100% sensitive nor have the same sensibility; in addition, the development of the antibodies could be delayed [105].

The presence of other clinical features suggesting the involvement of other systems in conjunction with cerebellar ataxia is essential clinical evidence and can guide the physician towards the antibody of interest and the possible underlying malignancy [106]. For example, isolated rapidly progressive cerebellar ataxia is linked to anti-Yo, -DNER-(Tr/delta/notch-like epidermal growth factor-related receptor), and -mGluR1-antibodies [106]. Yo-antibodies are mainly associated with ovarian or breast cancer, DNER-antibodies with Hodgkin lymphoma (detected in 80% of patients), and mGluR1-antibodies with hematologic tumours (detected in about 30% of patients) [17]. Symptoms suggesting a more diffuse involvement of the central nervous system (encephalomyelitis) are associated with Hu-antibodies and the presence of small cell lung cancer; symptoms such as laryngeal spasm and opsoclonus in association with PCD are associated with Ri-antibodies and ovarian, breast, or small cell lung cancer [107].

Ultimately, paraneoplastic neurological syndromes (PNSs) are rare and difficult to diagnose; as said before, the diagnosis is based on excluding other pathologies. However, in November 2002, an international group of neurologists began to establish guidelines to provide more accurate criteria for diagnosing PNSs [97]. According to these criteria, PNSs can be divided into two main groups: classical and non-classical PNSs. Classical syndromes are often associated with cancer, and their discovery should trigger the search for the tumour regardless of ONA presence [4]. To define the cerebellar syndrome as “classical”, it should meet some characteristics: (i) development in less than 12 weeks of a severe pancerebellar syndrome with no MR evidence of cerebellar atrophy other than that expected by the age of the patient; (ii) the severity of the cerebellar syndrome should cause symptoms which have a significant negative impact on lifestyle (Rankin score of at least 3); (iii) in the first stage of the syndrome, gait ataxia may be the main or the only element present, but signs of truncal and hemispheric cerebellar dysfunction are required for the diagnosis.

Eventually, although clinical signs of cerebellum involvement are peculiar, the presence of other symptoms/signs suggesting the involvement of other parts of the central and peripheric nervous system is not uncommon and does not rule out the diagnosis [16].

### 3.3. Paraneoplastic Neuropathies

Paraneoplastic neuropathies constitute a pathological subset of the paraneoplastic neurologic syndrome, characterised by symptomatic manifestations and clinical signs due to injury of the nervous system not localised near the primitive cancer or its metastatic lesions [4,108,109].

Recognising different etiopathogeneses of peripheral neuropathy in a neoplastic patient is essential since neurological symptoms could indicate an early diagnosis of a tumour or a possible recurrence. Furthermore, distinguishing between the cause of neuropathy such as neoplasm infiltration, treatment induced, or paraneoplastic genesis provides the ability to undertake the most suitable treatment, preventing irreversible disabilities.

There are several differential diagnoses to consider like hyperviscosity, malnutrition, infections, amyloidosis, tumour infiltration of nerve structures, and iatrogenic consequences of chemotherapy and radiotherapy [108,110,111].

Discerning paraneoplastic neuropathy from the chemo-induced one constitutes a troublesome problem; however, it would be desirable to make a definite diagnosis since therapeutic regimens are profoundly different.

#### Classification of Neuropathies

Paraneoplastic neuropathies may be marked by a different pattern, progression, or the absence or presence of paraneoplastic antibodies; in this case, they could be distinguished between onconeural (also known as intracellular) and neuronal surface antibodies (also called NSAbs) (Table 3).

According to the clinical presentation, paraneoplastic neuropathies may be subdivided into generalised neuropathies (with possible sensory, motor, sensorimotor, or autonomic involvement), rarer and disputed entities, and focal nerve lesions (Table 2).

The subacute sensory neuronopathy represents the prototype of all the peripheral neuropathies, and inflammation in the dorsal root ganglia, associated with degeneration of the posterior column, plays a crucial role in the etiopathogenesis of the disease. In addition, CD8-positive cytotoxic T cells are responsible for the injury of sensory neuronal cells [113].

The clinical manifestations of the disease are characterised by asymmetric upper extremities involvement and painful dysesthesia or mechanical hyperalgesia without sensory ataxia due to significant affection of small neuron lesions [114].

Although there is no deficiency in muscular strength, the peculiar clinical feature is sensory ataxia, responsible for significant disability, with a corresponding electrophysiological finding of absent sensory responses and possible minimal alterations of motor responses [115].

The disease manifests itself with an acute/subacute onset and a rapid progression up to a plateau phase with a little or no chance of amelioration. In addition, it could be correlated with autoimmune pathologies such as Sjogren’s syndrome or idiopathic ones; however, considering that it is frequently paraneoplastic, excluding primitive malignancy, especially small cell lung cancer, is mandatory. It is possible to detect anti-Hu antibodies [29,116] or other antibodies like anti-CV2 (CRMP-5) or amphiphysin antibodies in the serum and cerebrospinal fluid.

Patients with serologic detection of anti-CV2 antibodies are more likely to present a clinical picture characterised by motor involvement and demyelination than those with anti-Hu antibodies [117]; nevertheless, paraneoplastic subacute sensory neuropathies could be seronegative in 16% of patients.

It could also be associated with other paraneoplastic syndromes like brainstem involvement, paraneoplastic encephalomyelitis, cerebellar degeneration, limbic encephalitis, motor neuropathy, and Lambert–Eaton myasthenic syndrome. In addition, some patients may be affected by autonomic dysfunction and, rarely, from myositis [118].

Pure sensorial neuropathy is a generalised neuropathy characterised by an insidious onset, sensory symptoms in the conventional glove-stocking distribution, incoordination, and neuropathic pain [119]. It occurs in autoimmune diseases and metabolic and toxic neuropathies and is not explicitly related to a paraneoplastic aetiology.

In case of the implication of both motor and sensory symptoms, neuropathy is defined as sensorimotor; therefore, it could constitute a paraneoplastic condition with eventual onconeural antibodies association [119,120], although it is often related to diabetes, alcohol, and chronic idiopathic axonal polyneuropathy in patients over 55 years.

The paraneoplastic sensory neuromyopathy usually occurs in advanced neoplastic patients with symmetric sensorimotor involvement, muscular weakness in distal and proximal muscles, and possible association with type 2 fibre muscle atrophy. Because it is not specific to cancer, it is necessary to rule out other concomitant factors such as weight loss, diabetes, and iatrogenic effects of chemotherapy.

Terminal neuropathy indicates mild sensorimotor neuropathy, which manifests itself in the advanced stages of several diseases, including the neoplastic one.

Lower motor neuropathy indicates a rare neuropathy characterised by generalised flaccid paresis with sparing of bulbar muscles and long tracts with subacute onset [121]; it may occur as a sequel of myeloma, plasma cell dyscrasia, other hematologic tumours, and after local radiotherapy.

Multiplex neuropathy is infrequently associated with paraneoplastic syndromes because it is more often related to vasculitis.

Myeloneuropathy presents as a major clinical factor of the a concomitant involvement of peripheral nerves and spinal cord ascribable from an etiopathogenetic point of view to a deficit of copper or vitamin B12 and toxic, infectious, and inflammatory diseases. Patients manifest motor and sensory signs such as impaired proprioception and paresthesias with the subacute onset and asymmetric distribution. They are often affected also by bladder dysfunction and pain. In the literature, Shah et al. [122] described a correlation between several antibodies and malignancies, particularly breast adenocarcinoma and SCLC.

Autonomic neuropathy can be observed in many diseases like AL amyloidosis, diabetes, autoimmune-related neuropathies, and paraneoplastic syndromes. The symptomatology is constituted by sweating abnormalities, urinary symptoms, orthostatic hypotension, gastroenteric dysmotility, intestinal pseudo-obstruction, and subacute dysautonomia.

Other rare and disputed entities are small fibre neuropathy, immune-mediated neuropathies, cryoglobulinemia, hyperexcitability syndromes, plasm cell dyscrasia, and paraproteinemia [123].

Paraneoplastic neurological syndrome could rarely be focal and imply cranial nerve injury, plexopathies, and muscular involvement. The cranial nerves most frequently affected are the II pair (optic n.), the V pair (trigeminal n.), and the VIII pair (vestibular n.); lesions of the oculomotor nerve are epidemiologically less common.

Concerning plexopathies, it is described as the involvement of cervical plexus (especially of the phrenic nerve), whereas among mononeuropathies in the literature, many cases of ulnar and peroneal nerves are reported.

In approximately 30% of patients suffering from chronic neuropathy, there is an iatrogenic etiopathogenesis, which is chemo-induced (CIPN, chemotherapy-induced peripheral neuropathy) with a dose-dependent probability of development. It is often associated with intravenous infusion of oxaliplatin and cisplatin in patients affected by breast or colon cancer [124].

### 3.4. Paraneoplastic Dermatomyositis

Dermatomyositis (DM) is an inflammatory disease that typically affects muscles and skin; it is supposed to be an autoimmune pathology, but the exact aetiology is still unclear.

Autoantibodies can be detected in half of patients and anti-Jo-1, directed against histidyl-tRNA synthetase, is the most common [125]. DM has two incidence peaks in childhood (5–15 years of age) and adulthood (40–60 years old).

#### 3.4.1. Clinical Features of DM

The muscular involvement is characterised by symmetric and proximal weakness in the first phases; later, neck muscles can be affected, and it is possible to observe dysphonia, dysphagia, and dyspnoea due to the injury to the muscles that take part in respiration.

Cutaneous findings associated with dermatomyositis include Grotton’s papules (pink-violaceous papules over the interphalangeal and metacarpophalangeal joints), heliotrope eruption with or without oedema in the periorbital skin, and additional features, such as violaceous erythema of the scalp, V-sign of the extensor surfaces of the upper extremities, neck, chest and upper back. The onset of cutaneous symptomatology can precede the appearance of myositis by up to several months or follows shortly after muscle involvement.

Another relevant and common (nearly 40% of the cases) feature of DM is lung impairment characterised by an interstitial disease, leading to fibrosis and consequent pulmonary hypertension [126].

#### 3.4.2. Pathophysiology of DM

DM is characterised by T and B cells, plasma cells, and macrophages in the endomysium [127]; the primary target is the endothelium of the endomysial capillaries, which is harmed by C3b, C3bNEO, C4b, and C5b-9. In the muscle biopsy, several patterns can be detected, including loss of capillaries and deposits of C5b-C9 on the capillaries [126,128].

As far as it concerns the skin, it is possible to observe apoptosis of the basal epidermal cells and increased mucin in the derma [129].

#### 3.4.3. Diagnosis of DM

Electromyogram (EMG) is sensitive but shows non-specific findings, such as early recruitment, positive sharp waves, and fibrillation potentials [130].

Magnetic resonance imaging (MRI) can detect muscular inflammation characterised by hyperintensity on T2-weighted scans [131].

Furthermore, muscular weakness is associated with increased levels of aspartate and alanine aminotransferase (AST/ALT), creatine kinase (CK), and lactate dehydrogenase (LDH).

Subjects with both muscular and cutaneous involvement are affected by classic dermatomyositis. A subset of patients develop skin disease with no muscular symptoms; in this case, the disease is defined as amyopathic DM. If the muscle impairment is only detectable with laboratory imaging and electrophysiologic techniques without symptomatology, DM is called hypomyopathic DM [132]. The presence of muscular involvement without cutaneous findings is defined as polymyositis (PM).

An increased malignancy rate has been described in subjects with DM, but the specific link is still not entirely understood [81].

#### 3.4.4. Association between DM and Cancer

Paraneoplastic dermatomyositis is a distinct subtype of dermatomyositis in which the specific cutaneous disease and muscle weakness appear before, simultaneously, or after the diagnosis of a malignancy. Different neoplasm have been associated with DM, especially lung, ovaries, pancreas, bladder, and stomach cancers, as well as hematologic cancers including non-Hodgkin and Hodgkin’s lymphoma [133].

This association is enhanced by the evidence of a temporal relationship between the diagnosis of cancer and myopathy; in addition, an improvement of myopathy can be observed after the cancer treatment.

The risk of cancer development in DM is five times higher than in the general population, with an increased risk of malignancy before and after the onset of the muscular symptomatology [134]. Higher incidence has been observed during the first year after the diagnosis of myopathy and fell gradually after five years of follow-up [135]. This observation could suggest a possible paraneoplastic aetiology.

The risk of cancer is more significant in patients with DM than in those with PM, and risk factors associated include severe cutaneous disease (capillary damage on muscle biopsy, cutaneous necrosis), older age of onset, dysphagia, and interstitial lung disease [136,137],

Moreover, the presence of some serum antibodies, such as anti-transcriptional intermediary factor 1 antibody (anti-TIF1-Ab), anti-p155, and antibodies against nuclear matrix protein (NXP)-2, anti-MJ, or anti-p140, are correlated with an increased risk of cancer [138,139,140].

In patients with anti-TIF1-Ab, the most common neoplasm associated seems to be breast cancer, followed by ovarian and lymphoma.

On the other hand, detecting myositis-specific antibodies (anti-Mi-2, anti-SRP, anti-synthetase Ab) and myositis-associated antibodies (anti-RNP, anti-Ku, anti-PM-Slc) is associated with a decreased risk of malignancy and an increased risk of interstitial lung disease [139].

Although the mechanism of the association between DM and neoplasm remains uncertain, a possible explanation could be represented by the expression of common antigenic myositis specific autoantigens in both tumour cells and undifferentiated myoblast.

Once the diagnosis of DM is established, clinicians should exclude the presence of malignancy with first-level exams such as laboratory tests (complete blood count, liver function, faecal occult blood test) and instrumental examinations like chest radiograph, mammography, and colonoscopy. Second-level exams like computed tomography (CT) of the chest/abdomen/pelvis should be recommended in those patients with high risk of underlying cancer. Follow-up should be continued for at least five years [81].

### 3.5. Chronic Gastrointestinal Pseudo-Obstruction

Chronic Intestinal Pseudo-Obstruction (CIPO) is a rare disorder characterized by impaired gastrointestinal propulsion with symptoms and objective signs of mechanical bowel obstruction without x-ray evidence of this element [141]. The first step in the diagnostic algorithm of CIPO is the execution of an abdominal X-ray, which is the simplest way to determine the presence or the absence of mechanical obstruction. In 85% of patients, there is a fluid–gas plane and general dilatation of the intestine. Gas accumulation is expected in the colon, while the mechanical ileus is in the distal portion of the intestine [142].

Although CIPO is more frequently idiopathic, different aetiologies could cause this condition (secondary causes were identifiable in only 4 cases out of 77 (5%) in a study [143]), including neurological diseases (degenerative neuropathies such as those determined by Diabetes mellitus, Parkinson’s disease), immune-mediated diseases (e.g., Systemic sclerosis, Systemic Lupus Erythematosus, dermatomyositis), infectious diseases (e.g., Chagas), genetic diseases (e.g., Hirschsprung disease), radiation or chemotherapy sequalae, and paraneoplastic conditions [144,145,146].

Autoimmune paraneoplastic CIPO (AP-CIPO) has been associated with several solid tumours, more frequently with small cell lung cancer and carcinoid tumour [147,148], and also with anaplastic lung adenocarcinoma, retroperitoneal lymphoma, and ovarian papillary serous adenocarcinomas [148].

Gastrointestinal symptoms preceded the diagnosis of the malignancy in all cases of small cell lung cancer by a mean duration of 8.7 months, while the diagnosis of malignancy preceded GI symptoms in the other cases [143].

#### 3.5.1. Pathophysiologic Mechanism of AP-CIPO

Similarly to other paraneoplastic syndromes, the pathophysiologic mechanism responsible for developing AP-CIPO involves auto-antibody-mediated nervous system inflammation [141]. Histological features of CIPO include myenteric plexus infiltration with plasma cells and lymphocytes, which is linked to axonal and neuronal degeneration, thus leading to myenteric plexus dysfunction and gastrointestinal dysmotility [149,150]. Among onconeural antibodies, anti-Hu is more frequently associated with paraneoplastic CIPO, and its detection often precedes the manifestation of the underlying tumour, thus giving it a potential diagnostic and prognostic role [151]. However, so far, evidence cannot establish a relationship between antiHu titers and the severity of the disease [127,143]. Other frequently associated ONAs are Purkinje cell cytoplasmic type 1 (anti-Yo) and N-type voltage-gated calcium channel antibodies [145], together with type 2 anti-neuronal nuclear antibody (anti-Ri), amphiphysin antibody, PCA-2, and CRMP antibody, although the latter is less frequently associated with this condition [152].

#### 3.5.2. Signs and Symptoms of AP-CIPO

As for specific clinical presentation (signs and symptoms), which could facilitate the differential diagnosis among the many possible ones, unfortunately, given the condition’s rarity, data have been limited to single-patient case reports and small case series. Pamarthy et al. [153] examined clinical aspects, imaging findings, and physiological study results (scintigraphic transit and manometry) in a large cohort of CIPO cases associated with scleroderma, amyloidosis, and paraneoplastic syndrome evaluated at a single tertiary referral centre. Among other findings, myopathic involvement was more common in systemic sclerosis, contrary to neuropathic involvement in paraneoplastic syndrome. Manometry is crucial in disease identification; low-amplitude contractions indicate a myopathic process, while uncoordinated contractility indicates a neuropathic process [154].

The GI dysfunction can manifest as gastroparesis, pseudo-obstruction, oesophageal achalasia, or other forms of dysmotility, depending on the interested GI tract.

### 3.6. Opsoclonus-Myoclonus Syndrome

Opsoclonus-myoclonus syndrome (OMS) is a neurologic disorder characterized by abnormal eye and axial/limbic movements, associated or not to cerebellar signs such as ataxia.

The term “opsoclonus” means the continuous, involuntary, uncontrolled, and arrhythmic ocular oscillation that occurs in all directions and without an intersaccadic interval; the multidirectional characteristic allows one to distinguish opsoclonus from ocular flutter shares, which are usually unidirectional. These abnormal eye movements are characterized by a high frequency (15 Hz) and large amplitude and appear during convergence eye movements or fixation.

The term “myoclonus” refers to the onset of rapid muscle spasms or a group of muscles involving different body regions (neck, trunk, limbs).

Other clinical features of opsoclonus-myoclonus syndrome are ataxia, oscilloscopes, vertigo, sleep disorders, and behavioural disturbances. Though most cases present with characteristic opsoclonus and myoclonus, some patients could lack these clinical signs [155,156,157].

#### 3.6.1. Pathophysiology of Opsoclonus-Myoclonus Syndrome

The etiological substrate of OMS includes autoimmune pathways, as confirmed by the finding of autoantibodies in the CSF or the serum of most patients, primarily in those with paraneoplastic OMS. Antibodies directed against Ri (ANNA-2), Hu (ANNA-1), YO (PCA-1), NMDA receptor, amphiphysin, and neurofilaments are the most detected, even though most of the patients are seronegative. The anti-Ri type II anti-neuronal nuclear anti-body (ANNA-2) is associated with fallopian or breast cancer in adults [158,159], whereas OMS in lung cancer is frequently antibody negative or associated in a few cases with glycine receptor antibodies [160].

Furthermore, an increased expression of B-cell-related cytokines and B-cell-activating factor (BAFF) in the biological fluids of these patients may indicate the implication of cell-mediated immune mechanism, mainly B-cell activity [161,162]. The detection of an elevated CSF/serum BAFF ratio supports the intrathecal BAFF synthesis [162,163].

The autoimmune genesis of OMS is supported by evidence of good response to corticosteroid and immunosuppressive therapy (i.e., rituximab), as well as by familial association to other autoimmune diseases such as thyroid diseases or rheumatoid arthritis [164,165,166].

#### 3.6.2. Brainstem Theory vs. Cerebellar Theory

Even though there are different proposed hypotheses, the pathogenetic mechanism responsible for opsoclonus is unknown. It may be caused by reduced inhibition/excess of activation of omnipause neurons in the nucleus raphe interpositus in the pons (brainstem theory) or by a failure in inhibition of the cerebellum fastigial nuclei (cerebellar theory) [41,42,43].

According to brainstem theory, saccadic movements originate after activation of burst neurons of the rostral interstitial nucleus of Cajal and paramedian pontine reticular formation, generally inhibited by omnipause neurons (OPNs). Based on this physiologic mechanism, the inefficacy of OPNs in inhibiting burst neurons may determine opsoclonus [41,167,168].

The cerebellar theory assumes that opsoclonus may be due to abnormal activation of the fastigial nucleus due to a lack of inhibitory projection from the dorsal vermis in the cerebellum [152,169,170].

#### 3.6.3. Diagnosis of Opsoclonus-Myoclonus Syndrome

Opsoclonus-myoclonus syndrome typically affects adult subjects. In most cases, OMS is idiopathic, even if up to 40% of cases in adults may be related to the neoplastic disease: in this case, small cell lung cancer is the most often diagnosed tumour. Other tumours associated with OMS are breast cancer, ovarian teratoma, gynaecologic cancer, gastric adenocarcinoma, and malignant melanoma [107,171,172,173,174,175,176,177,178,179]. In addition, the literature reports post-infectious cases in association with viral (rotavirus, hepatitis C, enterovirus, West Nile virus, EBV, HIV, CMV) and bacterial (salmonella, streptococcus, Mycoplasma pneumoniae, Rickettsia conorii) infection [180,181,182,183,184,185,186,187,188,189]. Rare cases are pregnancy related [190,191].

Sun-Yong Oh et al. proposed four criteria for diagnosing this rare syndrome: the presence of opsoclonus, presence of myoclonus or ataxia, behavioural changes and sleep disturbances, concomitant neoplasm or presence of antineuronal antibodies. For diagnosis, three of four criteria must be fulfilled [192].

### 3.7. Peripheral Nerve Hyperexcitability Syndromes

Peripheral nerve hyperexcitability (PNH) syndromes include several manifestations resulting from abnormal discharges of peripheral axons. The abnormal nerve activation leads to irregular muscle contractions determining various clinical features, such as myokymia, neuromyotonia, or fasciculations.

Myokymia results as continuous undulating muscle movements, flowing with a series of small waves on the surface described as “underskin wriggling snake”; electromyographic study is characterized by spontaneous and irregular discharges involving multiple single motor units, with a variable frequency (range from 5 to 150 Hz) [193].

Neuromyotonia manifests with prolonged muscle contraction in the absence of relaxation; it derives from high-frequency (150–300 Hz) activation of a single myofiber as a consequence of stimulation (nerve percussion, ischemia, movement) [194]. Electromyographic characteristics of neuromyotonia are the rhythmic repetition of high-frequency (150–300 Hz) multiple bursts and amplitude reduction at the beginning of the discharge [193].

Other non-specific clinical features are fasciculations, described as non-continuous muscle contractions and cramps; these usually are related to a benign syndrome called cramp-fasciculation syndrome.

#### 3.7.1. Pathologic Substrates of PNH Syndromes

As proposed by Isaacs [195], PNH syndromes may result from alterations in the terminal portions of the peripheral nerve. The lack of inhibition of muscle twitch after general anaesthesia or injection of anaesthetics having an impact on peripheral nerve trunks supports this assumption; at the same time, we can observe a reduction in abnormal activities after administration of drugs acting in the presynaptic (i.e., botulinum toxin) or postsynaptic (i.e., curare) phase [196].

PNH syndromes are probably related to autoimmune mechanisms, as demonstrated by the improvement of symptoms and the resolution of EMG alterations after plasma exchange or corticosteroid treatment; the frequent association with other autoimmune diseases (myasthenia gravis, Hashimoto thyroiditis, Addison disease) supports this theory.

On average, 30–40% of patients affected by PNH syndromes show autoantibodies against voltage-gated potassium channels (VGKC); the inhibition of these channels, expressed in the axons and nerve terminals of central and peripheral nervous system neurons, provokes spontaneous depolarization with consequent muscular contraction [30].

High titers of VGKC antibodies are often associated with the expression of antibodies reacting against two proteins related to VGCK: LGI1 (leucine-rich glioma inactivated 1), which interacts with metalloprotease domains (presynaptic ADAM 23 and postsynaptic ADAM22), and CASPR2 (contactin-associated protein2). The latter is often associated with paraneoplastic PNH syndromes, especially in patients affected by thymoma, usually with a poor prognosis [31,197]. In addition, some subjects affected by cramp-fasciculation syndrome (CFS) can express anti-VGCK antibodies, but in most cases, the target antigen of the immune response is unknown.

Peripheral nerve hyperexcitability syndromes can be associated with some tumours, representing one of the most common paraneoplastic syndromes. PNH-related cancers are frequently thymoma and small cell lung cancer, while non-Hodgkin lymphoma, lymphoplasmacytic lymphoma, bladder cancer, ovarian cancer, and hemangioblastoma are less frequently associated [30,198,199,200].

Sometimes, gene mutations modifying the pathways involved in the depolarization of the neuronal membrane can determine hereditary PNH syndrome. In this context, the mutation of the HINT1 (histidine triad nucleotide-binding protein 1) gene in chromosome 5 is responsible for so-called autosomal recessive axonal neuropathy associated with neuromyotonia (ARAN-NM) [201].

Rare causes of secondary PNH syndromes are drugs, infections, peripheral neuropathy, and motor neuron diseases.

#### 3.7.2. Classification of PNH Syndromes

Isaacs’ syndrome is the most well-known PNH syndrome and mainly affects middle-aged men. Neuromyotonia characterizes this syndrome and determines generalized stiffness and observable myokymia in at least two skeletal regions, mainly in the lower, rather than upper, limbs, although it can impact every part of the body, including the trunk and facial muscles. These manifestations are present in more than 90% of patients [202,203].

Sometimes, muscle contractions can lead to abnormal and painful postures and hinder walking; furthermore, the involvement of axial muscles can lead to hyperlordosis and respiratory failure.

Other clinical manifestations include painful cramps associated with muscle stiffness, typically worsening at rest and presenting at night, and continuous muscle contraction can lead to muscle hypertrophy and hyperhidrosis. Hyperirritability of the peripheral sensory nerves is associated with dysesthesias, paresthesias, or other sensory symptoms, frequently triggered by movements [204]. Hyporeflexia can be due to prolonged twitch and difficulty in relaxing.

Morvan’s syndrome appears with similar aspects to Isaacs’ syndrome. However, typical clinical features include neuropathic pain associated with electrophysiological evidence of neuropathy and frequent manifestations of central nervous system involvement, like amnesia, behavioural disturbances, and seizures [39,40]; almost all patients manifest hyperhidrosis, while other dysautonomic manifestations (tachycardia, hypertension, constipation, and diarrhoea) are less common [205]. A polymorphic syndrome including sleep disorders with loss of slow-wave sleep, abnormality of consciousness as stupor, alterations in the content of thought, such as hallucinations, and signs derived from motor and autonomic hyperactivation describe the “Agrypnia excitata”, typical of Morvan’s syndrome [206,207,208,209].

In patients suffering from Morvan’s syndrome, brain resonance usually shows alterations in temporal lobes; instead, functional tests underline decreased metabolism in the orbitofrontal, anterolateral temporal, and left medial temporal regions [210,211,212]. On the contrary, instrumental investigations are usually regular in Isaacs’ syndrome.

Cramp-fasciculation syndrome (CFS) is considered a benign syndrome whose main manifestations are fasciculations, cramps, and occasionally myokymia, usually associated with electromyographic abnormalities less severe than Isaac’s or Morvan’s syndromes [30,213].

Some rarer disorders can present with clinical manifestations similar to PNH syndromes, and clinicians often must pose differential diagnoses: episodic ataxia type 1, presenting with myokymia and generalized ataxia; Schwartz–Jampel syndrome, characterized by skeletal deformities, facial dysmorphism, and myotonia; rippling muscle syndrome, in which muscle percussion provokes rolling movements; and Stiff-person syndrome, in which prolonged muscle contractions lead to generalized stiffness [214,215,216,217,218,219,220,221,222,223,224].

Lambert–Eaton myasthenic syndrome (LEMS) is an uncommon disorder of neuromuscular junction transmission with a primary clinical manifestation of muscle weakness. Knowledge of subtle clinical features and laboratory abnormalities that accompany LEMS permits the early identification of the disorder. Early recognition is particularly important because of its strong association with small cell lung cancer (SCLC). Although LEMS can occur at any point in the course of SCLC, it may serve as a marker for early disease.

LEMS is a disorder of reduced acetylcholine (ACh) release from the presynaptic nerve terminals, despite normal ACh vesicle numbers, normal ACh presynaptic concentrations, and normal postsynaptic ACh receptors. Lambert and Elmqvist, through a series of elegant intracellular muscle recordings, found that patients with what is now called LEMS had the following unique features [1,2]:

- Normal miniature endplate potential amplitude, demonstrating normal postsynaptic sensitivity to ACh;
- Markedly reduced evoked endplate potential amplitude, suggesting a significant reduction in ACh release.

### 3.8. Therapy

The treatment of paraneoplastic neurological syndromes is closely related to several pathways that are pathogenically implied.

Commonly, regardless of paraneoplastic syndrome, it is strategically advantageous to detect a tumour and, where feasible, surgically remove it to improve neurologic symptomatology.

Unfortunately, since paraneoplastic syndromes are often related with onconeural antibodies, it is uncommon to resolve the clinical picture completely.

The use of immunomodulatory or immunosuppressive therapy in paraneoplastic cerebellar degeneration is based on limited data, and no controlled trial or even extensive series has been published. In the majority of reported patients, immunomodulatory and/or immunosuppressive treatment is often combined with therapy directed at the underlying tumour, making it difficult to ascertain the role of either intervention. Most patients have been treated with corticosteroids, intravenous immunoglobulins, and/or cyclophosphamide, singly or in combination.

Corticosteroids: With regard of corticosteroids treatment, regimens have varied among clinical studies, but, in general, two approaches have been used: administration of intravenous methylprednisolone, 1000 mg daily for 3–5 days, and use of prednisone at a dose of 60–80 mg daily for a period of time depending on its effect on the disease. Frequently, intravenous methylprednisolone is used initially, to be followed by oral prednisone with eventual taper. In some cases high-dose intravenous methylprednisolone has also been used in repeating courses.

Intravenous immunoglobulins: This therapeutic approach have been used successfully for myasthenia gravis, Lambert–Eaton myasthenic syndrome, and stiff-person syndrome. Its use in paraneoplastic cerebellar degeneration, however, is speculative. The mechanisms by which intravenous immunoglobulin G affects host immunity are not understood. Possible mechanisms include reduction in T cell proliferation and induction of lymphopenia; suppression of pro-inflammatory cytokines; induction of lymphocyte monocyte apoptosis; suppression of B cell differentiation; endogenous immunoglobulin synthesis; and modulation of anti-idiotypic networks involved in immune tolerance. A dose of 0.4 g/kg intravenously for 5 days to a total dose of 2 gm/kg is recommended. Courses of intravenous IgG can be repeated, if necessary, usually as a course of 1–2 gm/kg. Several IgG preparations are commercially available, and dosage regimens differ somewhat among them. In general, however, infusion should begin at a rate of 0.01–0.02 mL/kg body weight per minute for 30 min. If this is well-tolerated, then the rate may be gradually increased to a maximum of 0.08 mL/kg body weight per minute.

Rituximab: A chimeric monoclonal agent which consists of human IgG1 constant regions and murine variable regions. The agent is specific for CD20, a transmembrane protein which is expressed on pre-B lymphocytes and B cells but not on plasma cells. CD20 is important in B-cell activation, proliferation, and differentiation. Rituximab is currently approved for the treatment of B cell (non-Hodgkin’s) lymphoma and rheumatoid arthritis and has been found to be of value in treatment of multiple sclerosis. A standard dosage regimen for use of rituximab in paraneoplastic disorders has not yet been established. Treatment of neuromyelitis optica has employed regimens of 375 mg/M2 for up to four infusions given at 4-week intervals or, alternatively, 1000 mg infused twice with 2 weeks between infusions. Use in paraneoplastic cerebellar degeneration has involved a regimen of 375 mg/M2 for up to four infusions given at 4-week intervals.

As shown from many individual recommendations [225], immune-modulating therapies may hinder progression, mainly administered earlier than clinical onset. Therefore, the first therapeutic option is usually represented by steroids, followed by or in combination with plasma exchange and IVIG.

Plasma exchanges allow obtaining clinical amelioration, decreasing antibody titer, and decreasing electrical activities [226]. In non-responsive cases, anti-CD20 treatment or immunosuppressors like cyclophosphamide could be considered.

Immune checkpoint inhibitors (ICIs) have made an indelible mark in the field of cancer immunotherapy. Starting with the approval of anti-cytotoxic T lymphocyte-associated protein 4 (anti-CTLA-4) for advanced-stage melanoma in 2011, ICIs have obtained US Food and Drug Administration approval for the treatment of a wide array of cancer types, demonstrating unprecedented extension of patient survival. However, despite the success of ICIs, resistance to these agents restricts the number of patients able to achieve durable responses, and immune-related adverse events complicate treatment. Immune-checkpoint inhibitors in cancer treatment algorithms has renewed interest in PNSs. ICIs are associated with a considerably increased incidence of immunological toxicities compared with traditional anticancer therapies, including neurological immune-related adverse effects (nirAEs) that can manifest as PNSs. Theoretically, the use of ICIs might increase the risk of PNSs, in particular in patients with the types of cancer that are most frequently associated with these disorders (such as small cell lung cancer), emphasizing the importance of their prompt diagnosis and treatment to prevent irreversible neurological deficits [227].

Considering the potential for myelin regeneration, steroids or intravenous immunoglobulins can represent a successful strategy in demyelinating neuropathies; on the other hand, when axonal degeneration is present, response to immunotherapy depends on the degree of irreversible neuronal damage. In the case of sensory neuronopathy, sensorimotor neuropathies, and gastrointestinal pseudo-obstruction, often related to anti-Hu antibodies, patients may have stable but severe deficits in the council of the aggressiveness despite optimised medical treatment of the pathology.

Immunotherapy constitutes a winning strategy in patients affected by paraneoplastic autonomic neuronopathy, a ganglionopathy probably attributable to ganglionic AChR antibodies [228].

Unfortunately, the therapeutic aforementioned approaches are often less advantageous in antibody-mediated autoimmune encephalitis because they do not result in lower levels of intrathecal antibodies or decrease the antibody-producing cells in the central nervous system. In these patients, contemporary treatment advice refers to an experience with patients with anti-NMDAR encephalitis [229]. In about 50% of patients, there is evidence of response to initial corticosteroids and plasma exchange or IVIG; in the remaining 50%, more aggressive immunosuppression, like cyclophosphamide or rituximab, is needed. The anti-CD20 monoclonal antibody (rituximab) is effectively used in the treatment of B-cell lymphomas. Recent reports in the literature suggest that antibody-associated autoimmune disorders may respond to rituximab [230].

Furthermore, it is essential to undertake an appropriate symptomatic treatment to control pain and prevent disability and quality of life. This goal can be reached through antidepressants and GABA-mimetic drugs, which represent first-line therapy, and topical lidocaine and opioids, which represent second- and third-line drugs. In addition, antiepileptics drugs like lamotrigine, phenytoin, carbamazepine, and sodium valproate may be implied in neuromyotonia [112].

New investigations are necessary to gain updated scientific insights about the relation between cancer and immune and nervous systems to clearly define these therapies’ mechanisms and effects.

## 4. Conclusions

PNSs are a heterogeneous group of pathologies that represent a complex challenge for clinicians due to the variable and often non-specific symptomatology; since the neurological symptoms often precede cancer development, it is difficult to determine the paraneoplastic aetiology. Consequently, an early diagnosis of PNSs can heavily improve the prognosis of the neoplastic disease, potentially identifying the malignancy in its first stages. In addition, the discovery of new specific antibodies and the development of better techniques to identify them could help clinicians establish the correct diagnosis. The nervous system governs both ontogeny and oncology. Regulating organogenesis during development, maintaining homeostasis, and promoting plasticity throughout life, the nervous system plays parallel roles in the regulation of cancers. The direct paracrine and electrochemical communication between neurons and cancer cells and indirect interactions through neural effects on the immune system and stromal cells in the tumour microenvironment in a wide range of malignancies are worthy of future research. Nervous system–cancer interactions can regulate oncogenesis, growth, invasion and metastatic spread, treatment resistance, stimulation of tumour-promoting inflammation, and impairment of anti-cancer immunity.

## Figures and Tables

**Table 2:** Antibodies observed in subjects affected by limbic encephalitis.

Antibody	Neoplasm Associated
Anti-Hu	SCLC
CRMP-5 (collapsin response-mediator protein-5) [25,26].	Thymoma, SCLC
VGKC (voltage-gated potassium channel)	Various neoplasms
NMDAR [25,26].	Ovarian teratoma
Neuropil [25,26].	Thymoma, SCLC
Anti-Ma2 [21,22,23].	Testis
Amphiphysin [25,26].	SCLC, breast

**Table 3:** Classification of neuropathies [112].

Types Of Pn Neuropathies	Sub-Classification	Comments
**Generalised neuropathies**	Subacute sensory neuronopathy	Highly indicative of a PN cause, although also observed in other conditions
	Sensory neuropathy	Unspecific
	Sensorimotor neuropathy	Unspecific
	Sensory neuromyopathy and terminal neuropathy	Historic terminology
	Motor neuropathy	Rare
	Multiplex neuropathy	Vasculitis is rare
	Myeloneuropathy	Possibly new entity
	Autonomic neuropathies	Incidence uncertain
**Rarer and disputed entities**	Small fiber neuropathy	Incidence unclear, except for hematologically associated types
	Cryoglobulinemic neuropathy	
	Hyperexcitability syndromes	
	Paraproteinemia and AL amyloid	
**Focal nerve lesions**	Cranial nerves	Individual cases
	Nerve plexus	
	Mononeuropathies	

## Data Availability

Data sharing is not applicable to this article as no datasets were generated or analysed during the current study.
